# The Brody effect to detect hypovolemia in clinical practice

**DOI:** 10.1186/cc10839

**Published:** 2012-03-20

**Authors:** R Giraud, N Siegenthaler, DR Morel, K Bendjelid

**Affiliations:** 1 Hôpitaux Universitaires de Genève, Switzerland

## Introduction

The electrocardiogram (EKG) is a common monitoring method in intensive care medicine. Several studies suggest that changes in EKG morphology may reflect changes in volume status. The Brody effect, a theoretical analysis of left ventricular chamber size influence on QRS-wave amplitude, is the key element of this phenomenon. It is characterized by an increase in QRS-wave amplitude induced by an increase in ventricular preload [1]. This study investigated the influence of changes in intravascular volume status on respiratory variations of QRS-wave amplitude (EKG) compared with respiratory pulse pressure variations (PP).

## Methods

In 17 pigs, EKG and arterial pressure were recorded. QRS-wave amplitude was measured from the Biopac recording ensuring that in all animals EKG electrodes were always at the same location. Maximal QRS amplitude (EKGmax) and minimal QRS amplitude (EKGmin) were determined over one respiratory cycle. EKG was calculated as 100 × ((EKGmax - EKGmin)/(EKGmax + EKGmin)/2). EKG and PP were simultaneously recorded. Measurements were performed during normovolaemic conditions, after haemorrhage and following retransfusion with constant tidal volume (10 ml/kg) and respiration rate (15/minute).

## Results

At baseline, PP and EKG were both <12%. PP were significantly correlated with EKG (*r*^2 ^= 0.89, *P *< 0.001). Volume loss induced by haemorrhage increased significantly PP and EKG. Moreover, during this state, PP were significantly correlated with EKG (*r*^2 ^= 0.86, *P *< 0.001). Retransfusion significantly decreased both PP and EKG, and PP were significantly correlated with EKG (*r*^2 ^= 0.90, *P *< 0.001).

## Conclusion

Available correlations between PP and EKG at each time of the study were observed, meaning that EKG is a reliable parameter to estimate the changes in intravascular volume status and provide experimental confirmation of the Brody effect [2].
